# Hexokinase 2 Promotes Cell Growth and Tumor Formation Through the Raf/MEK/ERK Signaling Pathway in Cervical Cancer

**DOI:** 10.3389/fonc.2020.581208

**Published:** 2020-11-26

**Authors:** Nan Cui, Lu Li, Qian Feng, Hong-mei Ma, Dan Lei, Peng-Sheng Zheng

**Affiliations:** ^1^Department of Reproductive Medicine, The First Afﬁliated Hospital of Xi’an Jiaotong University, Xi’an, China; ^2^Key Laboratory of Environment and Genes Related to Diseases, Section of Cancer Stem Cell Research, Ministry of Education of the People’s Republic of China, Xi’an, China; ^3^Hebei Key Laboratory of Environment and Human Health, Department of Social Medicine and Health Care Management, School of Public Health, Hebei Medical University, Shijiazhuang, China

**Keywords:** HK2, cyclin A1, Raf/MEK/ERK, cervical cancer, proliferation

## Abstract

Hexokinase 2 (HK2) is a member of the hexokinases (HK) that has been reported to be a key regulator during glucose metabolism linked to malignant growth in many types of cancers. In this study, stimulation of HK2 expression was observed in squamous cervical cancer (SCC) tissues, and HK2 expression promoted the proliferation of cervical cancer cells *in vitro* and tumor formation *in vivo* by accelerating cell cycle progression, upregulating cyclin A1, and downregulating p27 expression. Moreover, transcriptome sequencing analysis revealed that MAPK3 (ERK1) was upregulated in HK2-overexpressing HeLa cells. Further experiments found that the protein levels of p-Raf, p-MEK1/2, ERK1/2, and p-ERK1/2 were increased in HK2 over-expressing SiHa and HeLa cells. When ERK1/2 and p-ERK1/2 expression was blocked by an inhibitor (FR180204), reduced cyclin A1 expression was observed in HK2 over-expressing cells, with induced p27 expression and inhibited cell growth. Therefore, our data demonstrated that HK2 promoted the proliferation of cervical cancer cells by upregulating cyclin A1 and down-regulating p27 expression through the Raf/MEK/ERK signaling pathway.

## Introduction

Oncogenic types of human papillomaviruses (HPVs) are closely linked to almost 5% of the total incidence of human cancers. Cervical cancer has been reported to be one of the most prevalent HPV-induced malignancies. Human papillomavirus (HPV) can be detected in almost 99.7% of cervical carcinoma cases, and it accounts for more than 250,000 cancer deaths and more than 500,000 new cancer cases worldwide every year (1, 2). HPV16 (55–60%) and HPV18 (10–15%) are high-risk HPV genotypes within cervical cancer (3), and these HPV sequences regularly continuously express the viral E6/E7 oncogenes, which are considered crucial for malignant cell transformation and the maintenance of cell growth (4).

Metabolic alteration is commonly present in cancer cells to support malignant growth by promoting cellular proliferation and cell survival (5). During metabolic reprogramming, most cancer cells reprogram cellular glucose metabolism to fulﬁll their anabolic demands. In cancer cells, the energy (ATP) that is mainly generated by aerobic glycolysis to support malignant growth is called the “Warburg effect” (6, 7). Hexokinase 2 (HK2) is a member of the hexokinases and was reported to be a key regulator in and to play a rate-limiting role during glucose metabolism (8). Generally, HK2 expresses at only very low concentrations in most normal tissues (e.g., skeletal, cardiac muscle and adipose tissues), but it is highly expressed in many malignant cells and tissues (6, 9). Previous studies have demonstrated that HK2 is required for tumor initiation and maintenance and is linked to tumor metastasis and growth in many types of cancers (6, 7, 10, 11). In cervical cancer, increased HK2 expression in cervical cancer samples compared to normal cervical tissues was already reported as early as the 1970s (12, 13). Recently, studies have demonstrated that ectopic overexpression of E7 stimulated HK2 expression in cervical cancer cell lines linked to radiation resistance (14), and it played a role as a negative prognostic marker in cervical cancer patients (15, 16). However, the evidences that about the molecular regulation mechanism of HK2 on regulating cell proliferation and tumor formation in cervical cancer cells remains not a lot.

Extracellular signal–regulated kinases 1 and 2 (ERK1/2) are viewed as canonical mitogen-activated protein kinases (MAPKs) involved in signal transduction and the regulation of transcription (17). ERK1/2 are activated through phosphorylation by upstream MAPK/ERK kinases MAPK/ERK kinase 1 and 2 (MEK1/2), which are activated through phosphorylation by upstream Raf serine/threonine protein kinases (Raf-1, B-Raf, and A-Raf) (18, 19). The activity of the Raf/MEK/ERK signaling pathway plays a redundant role in regulating fundamental biological processes, such as proliferation, survival, metastasis, and differentiation in many types of cancer, including cervical cancer (20–23). However, the potential links between HK2 and the Raf/MEK/ERK signaling pathway, which mediates cervical carcinoma initiation and growth, remain less known.

To address this issue, exogenous HK2 was stably over-expressed in cervical cancer cells. A transcriptome sequencing analysis was performed in HK2-overexpressing monoclonal cell lines to screen for potential target genes and signal transduction pathways that are likely involved in HK2-mediated cell growth and tumor formation in cervical cancer. As shown in this study, HK2 overexpression could activate ERK1/2 through the Raf/MEK/ERK signaling pathway, further promoting cell proliferation and tumor formation by inducing cyclin A1 and reducing p27 expression in cervical cancer cells.

## Materials and Methods

### Cell Lines and Human Tissue Specimens

Human cervical carcinoma cell lines SiHa, C-33A, HeLa, CaSki, and HT-3 were purchased from the American Type Culture Collection (ATCC, Rockville, MD, USA). High-glucose Dulbecco modified Eagle medium (DMEM, Sigma-Aldrich, St Louis, MO, USA) was used to culture SiHa, C-33A, and HeLa cells, RPMI1640 (Sigma-Aldrich, St Louis, MO, USA) was used to culture CaSki cells, McCoy’s 5A Medium (Sigma-Aldrich, St Louis, MO, USA) was used to culture HT-3 cells, and 10% fetal bovine serum (FBS; Hyclone, Thermo Scientiﬁc, Waltham, MA, USA) was added in all of the culture mediums.

A total of 39 squamous cervical cancer (SCC), 15 high-grade squamous intraepithelial lesion (HSIL), 16 normal cervixes (NC) tissues, normal cervix fresh samples, and squamous cervical cancers fresh samples were collected from the First Affiliated Hospital of Xi’an Jiaotong University during 2008 to 2018.

### Immunohistochemistry and Immunocytochemistry

The Immunohistochemistry and Immunocytochemistry used in this study performed as previously described (24). The intensity of staining was divided into four scores: 0 (no staining), 1 (light brown), 2 (brown), 3 (dark brown). The percentage of positive cells was divided into 5 scores: 0=<5%, 1 = 5% to 25%, 2 = 25% to 50%, 3 = 50% to 75%,4= >75%. The immunohistochemistry (IHC) score = percentage score × intensity score. HK2 staining in tissues was classified into two categories (negative and positive expression): a score of ≤1 was defined as negative, a score of ≥ 2 was defined as strong positive.

The antibodies used were as follows: anti-HK2 (1:200 dilution, sc-374091, Santa Cruz, USA), anti-cyclin A1 (1:250 dilution, sc-239, Santa Cruz, USA), anti-p27 (1:50 dilution, sc-1641, Santa Cruz, USA), anti-Ki67 (1:150 dilution, sc-23900, Santa Cruz, USA), anti-ERK1/2 (1:200 dilution, #4370, Cell Signaling Technology).

### Western Blotting

Western blotting analysis used in this study was performed as previously described (24). Antibodies against human HK2, cyclin A1, p27, p-Raf-1, MEK1/2, p-MEK1/2, ERK1/2, c-myc, GAPDH were purchased from Santa Cruz Biotechnology (Dallas, TX, USA). Anti-p-ERK1/2 was purchased from CST (Littleton, CO, USA). The horseradish peroxidase-conjugated anti-rabbit or anti-mouse IgG was purchased from Thermo Fisher Scientific (New York, NY, USA). The antibodies used were as follows: anti-HK2 (1:500 dilution, sc-374091, Santa Cruz, USA), anti-cyclin A1 (1:1,000 dilution, sc-239, Santa Cruz, USA), anti-p27 (1:300 dilution, sc-1641, Santa Cruz, USA), anti-MEK1/2 (1:500 dilution, sc-81504, Santa Cruz, USA), anti-p-MEK1/2 (1:500 dilution, sc-81503, Santa Cruz, USA), anti-ERK1/2 (1:500 dilution, sc-135900, Santa Cruz, USA), anti- p-Raf-1 (1:500 dilution, sc-271919, Santa Cruz, USA), anti-c-myc (1:500 dilution, sc-40, Santa Cruz, USA), anti-GAPDH (1:500 dilution, sc-47724, Santa Cruz, USA), anti-p-ERK1/2 (1:200 dilution, #4370, Cell Signaling Technology). GAPDH was used as the control and for quantification.

### Cell Growth Assays

Cell proliferation was detected by cell growth curves: 4 × 10^4^ cells were seeded in 6-well plates in triplicate, then cell number were counted every 2 days by using hemocytometer.

Cell viability was assessed using 3-(4,5-dimethylthiazole-yl)-2,5-diphenyl tetrazolium bromide (Sigma-Aldrich, St Louis, MO, USA) dye, and the absorbance value at 490 nm was detected by using plate reader.

### Tumor Xenograft Assay

The experimental protocols were evaluated and approved by the Animal Care and Use Committee of the Medical School of Xi’an Jiaotong University and the Ethics Committee of the First Affiliated Hospital of the Medical School of Xi ‘an Jiaotong University, and all of the animals were raised in a specific pathogen-free (SFP) room, with constant temperature (22–25°C) and humidity (40–50%). Twenty female BALB/c-nude mice were randomly divided into four groups, 1 × 10^5^ HK2 modified cervical cancer cells injected into the subcutis on the dorsum of each female BALB/c-nude mouse (4 to 6-week old, purchased from Slac Laboratory Animal Co., Ltd., Shanghai, China), and raising in a specific pathogen free (SFP) room. The mice were killed at the end of the experiment, all of the tumors that derived from HK2 modified cervical cancer cells was collected and weighed. Following formula was used to measure the tumor volume (V): V =ab^2^/2 (a: length, b: width).

### Flow Cytometry Analysis

FACS (BD Biosciences, San Jose, CA, USA) was used to analyze cell cycle in HK2 modified cervical cancer cells, and the data was analyzed by using the Cell-Quest software. 1×10^6^ HK2 modified cells in this study were washed with cold PBS for twice time, then fixing in cold ethanol (70%) at 4°C overnight. Next day, cells were washed with cold PBS for twice times, treating with RNaseA (Sigma-Aldrich, St. Louis, MO, USA) and staining with propidium iodide (Sigma-Aldrich, St. Louis, MO, USA).

### Real-Time PCR Analysis

Total RNA extractive and the protocol for real-time PCR was performed as previously described (24). The primer sequences that used in this study for real time PCR were shown in **Supplementary Table S2**. GAPDH was used as the house keeping gene in this study and all of the results were analyzed *via* the ΔΔCt method.

### DNA Construction and Transfection

Following primers: forward, 5’-CCGGAATTCGCCACCATGATTGCCTCGCATCTGCTTGCCTACT-3’ and reverse, 5’-CGCGGATCCCTATCGCTGTCCAGCCTCACG GATGC-3’ was used to amplify the full length of HK2. And the HK2 DNA fragment was cloned into pIRES2-AcGFP (Clontech, Mountain View, CA) with the EcoRI and BamHI sites. The short hairpin RNA (shRNA) for HK2 was purchased from Gene Pharma (Shanghai, China). Lipofectamine 2000 reagent (Invitrogen, Carlsbad, CA, USA) was used to transfect the pIRES2-AcGFP-HK2 and shRNA vectors into SiHa and HeLa cells to generate stably transfected cell lines by treating with G418 (Calbiochem, La Jolla, CA, USA) for 3 weeks.

### RNA Preparation and Transcriptome Resequencing

Total RNA of HeLa-GFP and HeLa-HK2 monoclonal cells were extracted by using TRIzol reagent (Invitrogen, Carlsbad, CA, USA) for transcriptome resequencing. And samples were measured using the BGISEQ-500 platform (The Beijing Genomics Institute, BGI), and the average output of each sample was 1.15Gb. The average ratio of sample to genome was 94.94%, and the ratio of comparison to gene set was 79.16%. The experiment analysis used the NOISeq method, which is a novel non-parametric approach for the identiﬁcation of differentially expressed genes (DEGs) based on log2 fold change>1 and a probability ≥0.80. Subsequent data analysis was performed by Dr. Tom on-line system from the Beijing Genomics Institute.

### Statistical Analysis

All of statistical analysis in this study was performed with GraphPad Prism 8.0 software and SPSS software version 19.0. Two-tailed unpaired Student’s t-test was used to determine the statistical significance for two-group analyses, and presented as mean ± SD.

*Post hoc* test was performed for comparison among groups. Chi-square test was used for count data. In all of the tests, statistical significance was defined as *p<0.05, **p<0.01, ***p< 0.001.

## Results

### The Expression of HK2 in Normal Cervixes, High-Grade Squamous Intraepithelial Lesion, and Squamous Cervical Cancer Cervical Lesions

**Figure 1:**
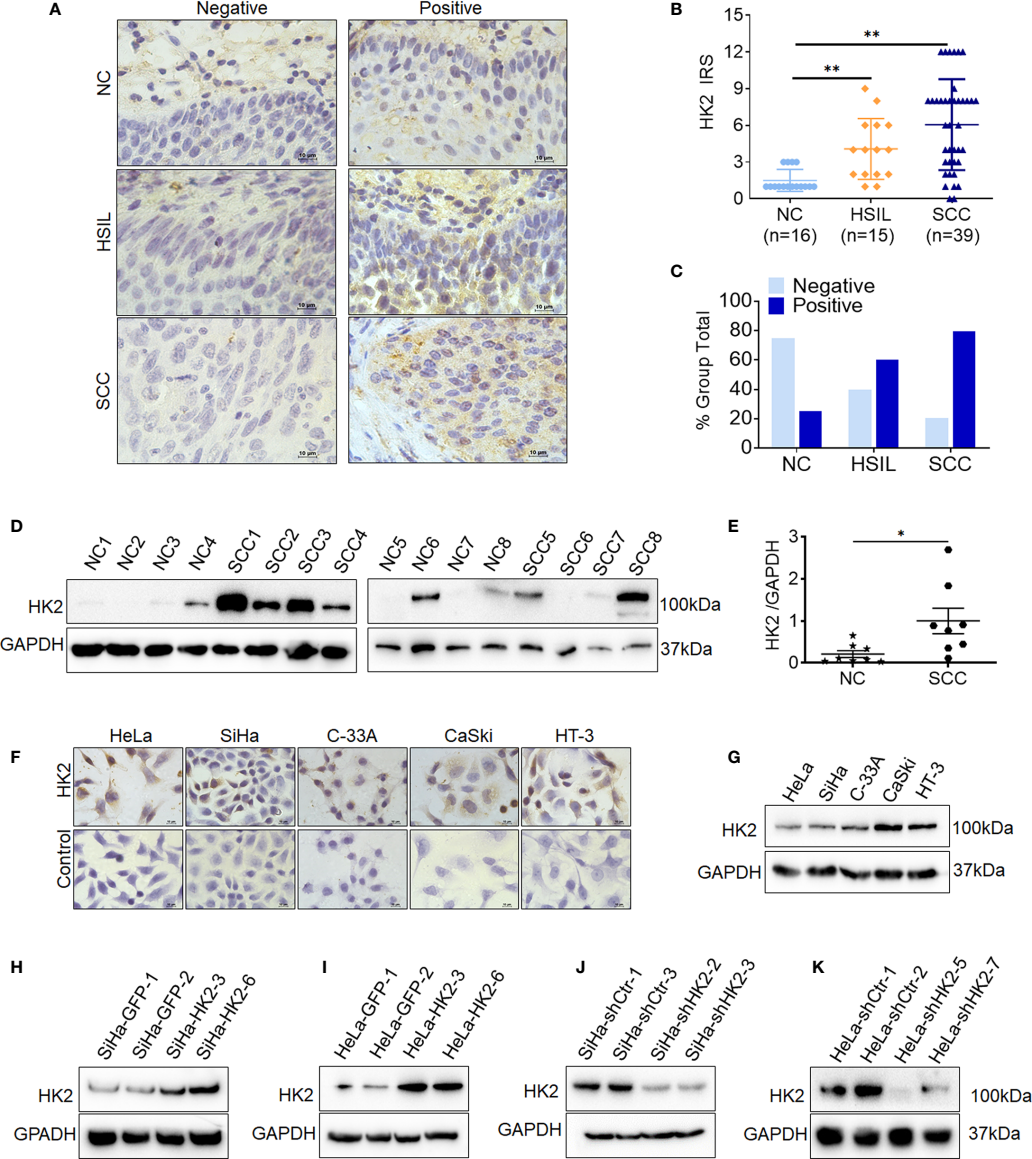
The expression of HK2 in NC, HSIL, and SCC cervical lesions. **(A)** Immunohistochemical (IHC) detection of HK2 in normal cervix samples (NC), high-grade squamous intraepithelial lesion (HSIL) and squamous cervical cancer lesions (SCC); original magnification, 1000×. **(B)** The scatter plot shows the immunoreactivity scores (IHC) obtained for the staining of HK2 in normal cervix, cervical cancer *in situ* and invasive cervical cancer samples (points represent the IHC score per specimen, and one-way ANOVA was performed). **(C)** HK2 stains is classified into negative and positive, and the bar chart shows the percentage of each group (16 NC specimens, 15 HSIL specimens, and 39 squamous cervical cancer specimens). **(D)** The expression of HK2 in eight normal cervix (NC) and eight squamous cervical carcinoma (SCC) samples was detected by western blot. **(E)** The relative expression of HK2 in each normal cervix tissue (n=8) and squamous cervical carcinoma tissue sample (n=8) is shown. The data shown are the ratios of the HK2/GAPDH of each specimen and the means ± standard error of the NC and SCC groups (triangles represent relative HK2 expression). HK2 expression in human cervical cancer cell lines was detected using immunocychemistry **(F)** and western blotting **(G)**. Stably transfected cell lines were identified by western blotting: **(H)** SiHa-GFP and SiHa-HK2 cells; **(I)** HeLa-GFP and HeLa-HK2 cells; **(J)** SiHa-shControl and SiHa-shHK2 cells **(K)** HeLa-shControl and HeLa-shHK2 cells. Values are shown as the mean ± SD, *p < 0.05, **p < 0.01.

To investigate whether HK2 is involved in the development and progression of human cervical carcinoma, immunohistochemistry (IHC) was used to detect HK2 expression in the normal human cervix (NC), high-grade squamous intraepithelial lesions (HSILs), and squamous cervical cancer (SCC) tissues. Representatively HK2 staining in the NC, HSIL, and SCC lesions was shown in **Figure 1A**. The average immunoreactivity scores were 2.06 ± 0.68 in NC, 4.20 ± 2.61 in HSIL, 6.23 ± 4.08 in SCC (**Figure 1B**, NC *vs.* HSIL, *p*<0.01; NC *vs.* SCC, *p*<0.01; HSIL *vs.* SCC, *p*=0.07). HK2 protein was localized in the cytoplasm; the positive rate was 25.00% in NC samples (4/16), 60% in HSIL samples (9/15), and 79.49% in SCC sample (31/39, **Figure 1C** and **Supplementary Table S1**). Additionally, the protein level of HK2 in eight cervical carcinoma samples and eight normal cervical samples was detected by western blotting (**Figure 1D**). The relative expression levels of HK2 in these cervical carcinoma samples were higher than that in the normal cervical samples (**Figure 1E**; *p*<0.05). All of these results suggested that HK2 expression was stimulated in cervical carcinoma tissues and might be involved in the process of cervical carcinogenesis.

Moreover, HK2 expression was observed in all five cervical cancer cell lines using western blotting and immunocytochemistry (HeLa, SiHa, C-33 A, CaSki, and HT-3, **Figures 1F, E**), and a relatively low expression of HK2 was observed in HeLa and SiHa cells. To further investigate the function of HK2 in human cervical cancer cells, exogenous HK2 was stably overexpressed in SiHa (SiHa-HK2, **Figure 1H**) and HeLa (HeLa-HK2, **Figure 1I**) cells; conversely, endogenous expression of HK2 was knocked down by stably transfecting shRNA plasmids in SiHa (SiHa-shHK2, **Figure 1J**) and HeLa (HeLa-shHK2, **Figure 1K**) cells.

### HK2 Promotes Proliferation of Cervical Cancer Cells *In Vitro*

**Figure 2:**
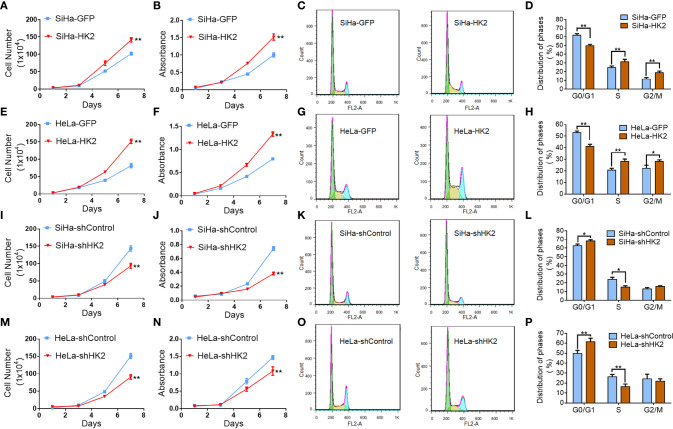
HK2 promoted cell proliferation of human cervical cancer cell lines *in vitro*. The cell proliferation in HK2 modified cells were detected by using growth curves: **(A)** SiHa-GFP and SiHa-HK2 cells; **(E)** HeLa-GFP and HeLa-HK2 cells; **(I)** SiHa-shControl and SiHa- shHK2 cells; **(M)** HeLa-shControl and HeLa-shHK2 cells. The cell viability in HK2 modified cells were detected by using MTT assay: **(B)** SiHa-GFP and SiHa-HK2 cells; **(F)** HeLa-GFP and HeLa-HK2 cells; **(J)** SiHa-shControl and SiHa- shHK2 cells; **(N)** HeLa-shControl and HeLa-shHK2 cells. The cell cycle was analyzed in HK2 modified cells by using flow cytometry: **(C)** SiHa-GFP and SiHa-HK2 cells and the quantitative analysis is shown in **(D)**; **(G)** HeLa-GFP and HeLa-HK2 cells and the quantitative analysis is shown in **(H)**; **(K)** SiHa-shControl and SiHa- shHK2 cells and the quantitative analysis is shown in **(L)**; **(O)** HeLa-shControl and HeLa-shHK2 cells and the quantitative analysis is shown in **(P)**. The data were shown as the mean ± SD of three independent experiments. *p < 0.05, **p < 0.01 vs. control using One-Way ANOVA.

Cell growth curves and MTT assay were applied to evaluate the proliferation and viability of HK2-modified cervical cancer cell lines and their control cells. Cell growth curves revealed that HK2 over-expression stimulated the proliferation of SiHa and HeLa cells (**Figures 2A, E**, P<0.05). MTT assay revealed that HK2 over-expression enhanced cell viability in SiHa and HeLa cells (**Figures 2B, F**, *p*<0.01). Moreover, both cell growth curves and cell viability assays revealed that HeLa-shHK2 and SiHa-shHK2 cells grew at much lower rates than their respective control cells (**Figures 2I, J, M, N**; *p*<0.01). All of these results demonstrated that the HK2 protein could promote the proliferation of cervical carcinoma cells *in vitro*.

Generally, the changes that occurred during cell proliferation involved the modulation of the cell cycle (25). Therefore, fluorescence-activated cells sorting (FACS) was used to analyze the differences in the cell cycle between the HK2-modified cells and their control cervical cancer cells. As shown in **Figures 2C, D**, HK2 over-expression caused a signiﬁcantly increased S phase (31.65 ± 2.64 *vs.* 25.18 ± 1.33, *p*<0.01) and G2/M phase (19.00 ± 1.68 *vs.* 11.93 ± 1.16, *p*<0.01) and a decreased G0/G1 phase (50.08 ± 1.34 *vs.* 60.10 ± 1.64, *p*<0.01) in SiHa-HK2 and SiHa-GFP cells. Similarly, HK2 over-expression caused a signiﬁcantly increased S phase (28.60 ± 1.61 *vs.* 20.90 ± 1.46, *p*<0.01), G2/M phase (28.53 ± 1.08 *vs.* 22.48 ± 2.45, *p*<0.01), and a decreased G0/G1 phase (41.30 ± 1.66 *vs.* 53.33 ± 1.06, *p*<0.01) in HeLa-HK2 and HeLa-GFP cells (**Figures 2G, H**). Conversely, the percentage of cells in G0/G1 phase was increased in both SiHa-shHK2 (**Figures 2K, L**, *p*<0.05, SiHa-shControl *vs.* SiHa-shHK2: 62.70 ± 1.98 *vs.* 68.27 ± 3.39) and HeLa-shHK2 cells (**Figures 2O, P**, *p*<0.01, HeLa-shControl *vs.* HeLa-shHK2: 49.66 ± 3.14 *vs.* 61.54 ± 1.35). The percentage of cells in S phase was decreased in both SiHa-shHK2 (**Figures 2K, L**, *p*<0.05, SiHa-shControl *vs.* SiHa-shHK2: 23.92 ± 2.45 *vs.* 15.02 ± 1.68) and HeLa-shHK2 cells (**Figures 2O, P**, *p*<0.01, HeLa-shControl *vs.* HeLa-shHK2: 26.32 ± 2.37 *vs.* 16.4 ± 2.62). All of these results suggested that this acceleration effect of HK2 on the cell cycle must be responsible for the stimulatory of cell proliferation in cervical cancer cells *in vitro*.

### HK2 Promotes the Growth of Cervical Cancer Cells *In Vivo*

**Figure 3:**
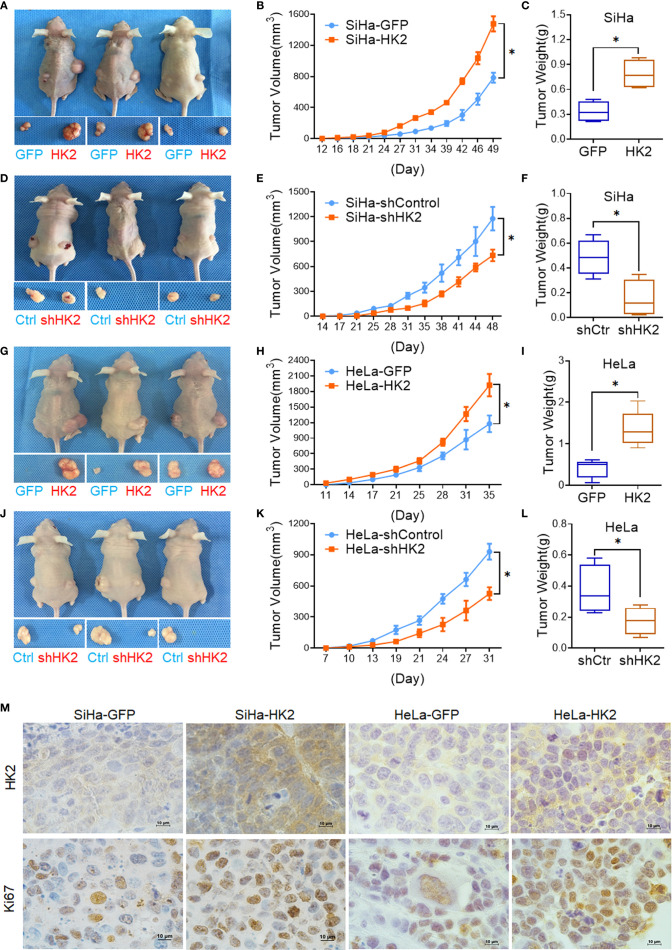
HK2 promoted cervical carcinoma tumor growth *in vivo*. Tumor growth curves were calculated after injection into female nude mice based on monitoring performed every 3 days: **(A, B)** SiHa-GFP and SiHa-HK2 cells; **(D, E)** SiHa-shControl and SiHa-shHK2 cells; **(G, H)** HeLa-GFP and HeLa-HK2 cells; **(J, K)** HeLa-shControl and HeLa-shHK2 cells. The xenograft tumors were dissociated and weighed at the end of experiment: **(C)** SiHa-GFP and SiHa-HK2 cells; **(F)** SiHa-shControl and SiHa-shHK2 cells; **(I)** HeLa-GFP and HeLa-HK2 cells; **(L)** HeLa-shControl and HeLa-shHK2 cells. **(M)** Immunohistochemical staining of HK2 and Ki67 in xenograft tumor tissues that derived from HK2 over-expressed SiHa and HeLa cells. Values are shown as the mean ± SD. *p < 0.05, *vs.* control using one-way ANOVA.

A total of 10^5^ HK2-modified cells and their control cells were inoculated subcutaneously into female nude mice for tumor formation assay to identify the effect of HK2 in cervical cancer cells *in vivo*. Xenografted tumors were successfully observed in both HK2-modified SiHa and HeLa cells (**Figures 3A, D, G, J**). The volume of the tumors formed by the SiHa-HK2 and HeLa-HK2 cells was much larger than their control cells (SiHa-GFP and HeLa-GFP, **Figures 3B, E**, *p*<0.05), and the weight of the tumors formed by the SiHa-HK2 and HeLa-HK2 cells was much heavier than that of the tumors formed by the SiHa-GFP and HeLa-GFP cells (**Figures 3C, F**, *p*<0.05). In addition, the tumors formed by SiHa-shHK2 and HeLa-shHK2 cells were much smaller (**Figures 3H, K**, *p*<0.05) and lighter (**Figures 3I, L**, *p*<0.05) than the tumors formed by SiHa-shControl and HeLa-shControl cells. All of these results demonstrated that the HK2 protein could promote tumor development of SiHa and HeLa cells *in vivo*.

Moreover, IHC revealed that the xenografted tumor tissues formed by SiHa-HK2 and HeLa-HK2 exhibited much stronger Ki67 (a cell proliferation marker) and HK2 stains than those formed by their control cells (SiHa-GFP and HeLa-GFP, **Figure 3M**, **Supplementary Figures S1A, C**). In addition, the tumor tissues derived from SiHa-shHK2 and HeLa-shHK2 cells expressed less Ki67 and HK2 than the tumor tissues derived from SiHa-shControl and HeLa-shControl cells (**Supplementary Figures S1B, D, I**). These results were consistent with the results obtained from the *in vitro* experiments in this study, suggesting that HK2 promoted the tumor growth of SiHa and HeLa cells *in vivo*.

### HK2 Altered the Expression of Cell Cycle-Related Proteins in Cervical Cancer Cells

**Figure 4:**
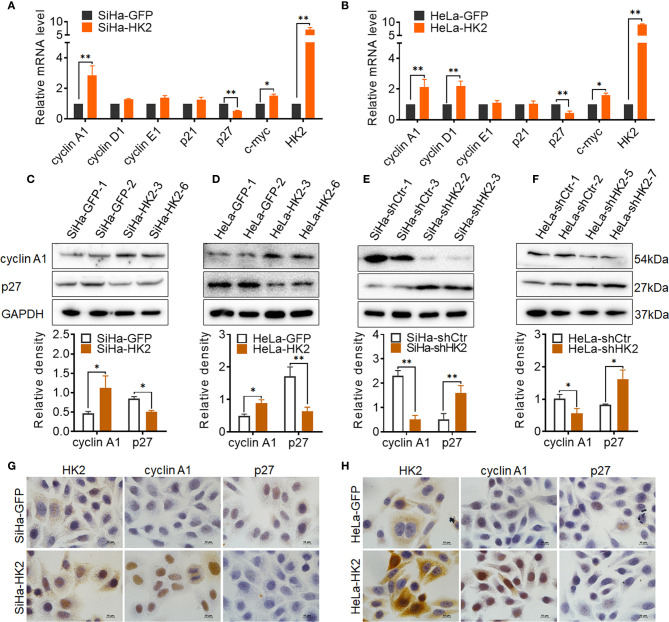
HK2 altered the expression of cyclin A1 and p27 in cervical cancer cells. **(A)** The messenger RNA (mRNA) expression of cell cycle related-proteins in SiHa-GFP and SiHa-HK2 cells was detected by real-time quantitative PCR and the quantitative analysis is shown. **(B)** The mRNA expression of cell cycle related proteins in HeLa-GFP and HeLa-HK2 cells was detected by real-time quantitative PCR and the quantitative analysis is shown. **(C)** The expression of cyclin A1 and p27 was detected by western blotting in HK2 modified cells and the quantitative analysis is shown: **(C)** SiHa-GFP and SiHa-HK2 cells; **(D)** HeLa-GFP and HeLa-HK2 cells; **(E)** SiHa-shControl and SiHa- shHK2 cells **(F)** HeLa-shControl and HeLa-shHK2 cells. **(G)** The expression of HK2, cyclin A1 and p27 in SiHa-GFP and SiHa-HK2 cells was detected by using immunocychemistry. **(H)** The expression of HK2, cyclin A1, and p27 in HeLa-GFP and HeLa-HK2 cells was detected by using immunocychemistry, original magnification, 1000×. Values are shown as the mean ± SD. *p < 0.05, **p < 0.01, *vs.* control using one-way ANOVA.

To explore the potential cell cycle-related proteins that are likely mediated by HK2 to promote cell growth in cervical cancer cells, real-time PCR, western blotting, and immunocytochemistry were applied to verify the expression of key cell cycle-related proteins in HK2-modified cells and their control cells. As shown in **Figures 4A, B** upregulated cyclin A1 mRNA levels and reduced p27 mRNA expression were observed in both SiHa-HK2 and HeLa-HK2 cells. In addition, increased cyclin D1 mRNA expression was also observed in HeLa-HK2 cells (**Figure 4B**, *p*<0.05). Moreover, the increased protein level of cyclin A1 and decreased protein level of p27 were observed in both SiHa-HK2 and HeLa-HK2 cells (**Figures 4C, D**, *p*<0.05). Conversely, the reduced protein level of cyclin A1 and induced p27 protein level were observed in HK2-knock-down cells (SiHa-shHK2 and HeLa-shHK2, **Figures 4E, F**, *p*<0.05). Moreover, cellular immunochemical analysis revealed that SiHa-HK2 and HeLa-HK2 cells showed much stronger cyclin A1 and weaker p27 stains than their control counterparts (**Figures 4G, H**, **Supplementary Figures S1E, F**). These results suggested that the acceleratory cell cycle progression mediated by HK2 likely depends on the up-regulated cyclin A1 and down-regulated p27 expression in cervical cancer cells.

### HK2 Activated ERKs (p-ERK1/2) in Cervical Cancer Cells

**Figure 5:**
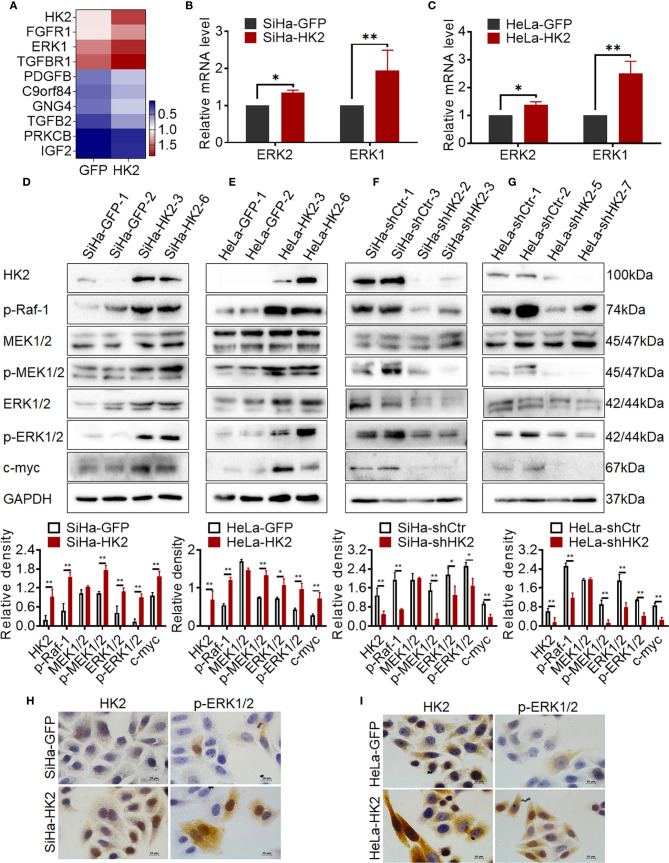
HK2 activated ERKs (p-ERK1/2) in cervical cancer cells. **(A)** Heatmap visualization of the differentially expressed genes that enriched in Ras/MAPK signaling pathway by Gene Ontology (GO) enrichment analysis through transcriptome sequencing between HeLa-HK2 and HeLa-GFP cell lines. Data were log^10^ normalized. **(B)** The messenger RNA (mRNA) expression of ERK1 and ERK2 was detected by real-time quantitative PCR and the quantitative analysis is shown: **(B)** SiHa-GFP and SiHa-HK2 cells, **(C)** HeLa-HK2 and HeLa-GFP cells. The expression of HK2, p-Raf-1, MEK1/2, p-MEK1/2, ERK1/2, p-ERK1/2, and c-myc was detected by western blotting, and the quantitative analysis is shown: **(D)** SiHa-GFP and SiHa-HK2 cells; **(E)** HeLa-GFP and HeLa-HK2 cells; **(F)** SiHa-shControl and SiHa- shHK2 cells **(G)** HeLa-shControl and HeLa-shHK2 cells. **(H)** The expression of HK2 and p-ERK1/2 in SiHa-GFP and SiHa-HK2 cells was detected by using immunocychemistry. **(I)** The expression of HK2 and p-ERK1/2 expression in HeLa-GFP and HeLa-HK2 cells was detected by using immunocychemistry, original magnification, 1000×. The data were shown as the mean ± SD of three independent experiments. *p < 0.05, **p < 0.01 *vs.* control using one-way ANOVA.

To further explore the potentially molecular mechanism by which HK2 altered the expression of cell cycle proteins in cervical cancer cells, a transcriptome sequencing analysis was performed in HeLa-HK2 and HeLa-GFP cell lines. A total of 210,503 transcripts were detected, and 258 up-regulated and 152 down-regulated genes were identiﬁed between the HeLa-HK2 and HeLa-GFP groups (**Supplementary Figure S2A**). The Ras/MAPK and PI3K-Akt signaling pathway was identiﬁed by KEGG pathway enrichment analysis (**Supplementary Figure S2C** and **Figure 2D**). There are 9 genes belonged to the Ras/MAPK signaling pathway and 12 genes belonged to the PI3K-Akt signaling pathway (**Figure 5A**, **Supplementary Figures S2B, D**). Unexpectedly, *MAPK3 (ERK1)*, a key factor involved in the Ras/MEKs/ERKs signaling pathway (26, 27), was significantly increased in the HeLa-HK2 group and was also presented in the gene list of both the Ras/MAPK and PI3K-Akt signaling pathways. Although *MAPK1 (ERK2)* was not identiﬁed by KEGG pathway enrichment analysis, the expression of *MAPK1* was also to some extent increased in the HeLa-HK2 group. Therefore, the mRNA levels of MAPK1 (ERK2) and MAPK3 (ERK1) in HK2-modified cells were confirmed by real-time PCR. As shown in **Figures 5B, C**, the mRNA levels of MAPK1 (ERK2) and MAPK3 (ERK1) were increased in HK2 over-expressing cells (HeLa-HK2 and SiHa-HK2). In addition, a mouse monoclonal antibody recommended for detecting the expression of both ERK1 and ERK2 in humans was used in this study. Consistent with the mRNA results, the protein level of ERK1/2 was increased in both HK2 over-expressing cells (SiHa-HK2 and HeLa-HK2, **Figures 5D, E**, *p*<0.05) and decreased in HK2 knock-down cells (SiHa-shHK2 and HeLa-shHK2, **Figures 5F, G**, *p*<0.05).

According to current research, activated ERK1/2 could translocate from the cytoplasm to the nucleus and participates in the biological response of cells depending on its phosphorylation form (p-ERK1/2) (28, 29). Therefore, it was attractive to detect the protein level of p-ERK1/2 in HK2 over-expressing cells. As shown in **Figure 5**, the protein level of p-ERK1/2 was upregulated in both SiHa-HK2 and HeLa-HK2 and down-regulated in HK2 knock-down cells (SiHa-shHK2 and HeLa-shHK2), suggesting that HK2 expression was positively correlated with ERK1/2 activity. Furthermore, immunocytochemistry confirmed increased immunochemical stains and nuclear translocation of p-ERK1/2 in SiHa-HK2 and HeLa-HK2 cells (**Figures 5H, I**, **Supplementary Figures S1G, H**, *p*<0.05). These data indicated that HK2 expression could up-regulate both total ERK1/2 and p-ERK1/2 expression in cervical cancer cell lines.

ERK is thought to be a downstream component activated by p-Raf and p-MEK1/2 (30); therefore, the protein levels of p-Raf-1, MEK1/2, and p-MEK1/2 were detected in HK2-modified cells by western blotting. As shown in **Figures 5D, E**, p-Raf-1 and p-MEK1/2 expressed much higher in HK2 over-expressing cells (SiHa-HK2 and HeLa-HK2) than in their control counterparts (SiHa-GFP and HeLa-GFP, **Figures 5D, E**, *p*<0.05). Furthermore, the p-Raf-1 and p-MEK1/2 proteins were at much lower levels in the SiHa-shHK2 (**Figure 5F**, *p*<0.05) and HeLa-shHK2 cells (**Figure 5G**, *p*<0.05), compared with their control cells. Moreover, p-ERK1/2 has been demonstrated to be an upstream regulator for maintaining c-myc expression. As shown in **Figure 5**, an increased protein level of c-myc was observed in HK2-overexpressing cells (SiHa-HK2 and HeLa-HK2, **Figures 5D, E**, *p*<0.05), and c-myc expression was reduced in HK2 knockdown cells (SiHa-shHK2 and HeLa-shHK2, **Figures 5F, G**, *p*<0.05). These results indicated that the activity of ERK1/2 in HK2-mediated cervical cancer cells likely occurred by phosphorylation through the Raf/MEK/ERK signaling pathway.

**Figure 6:**
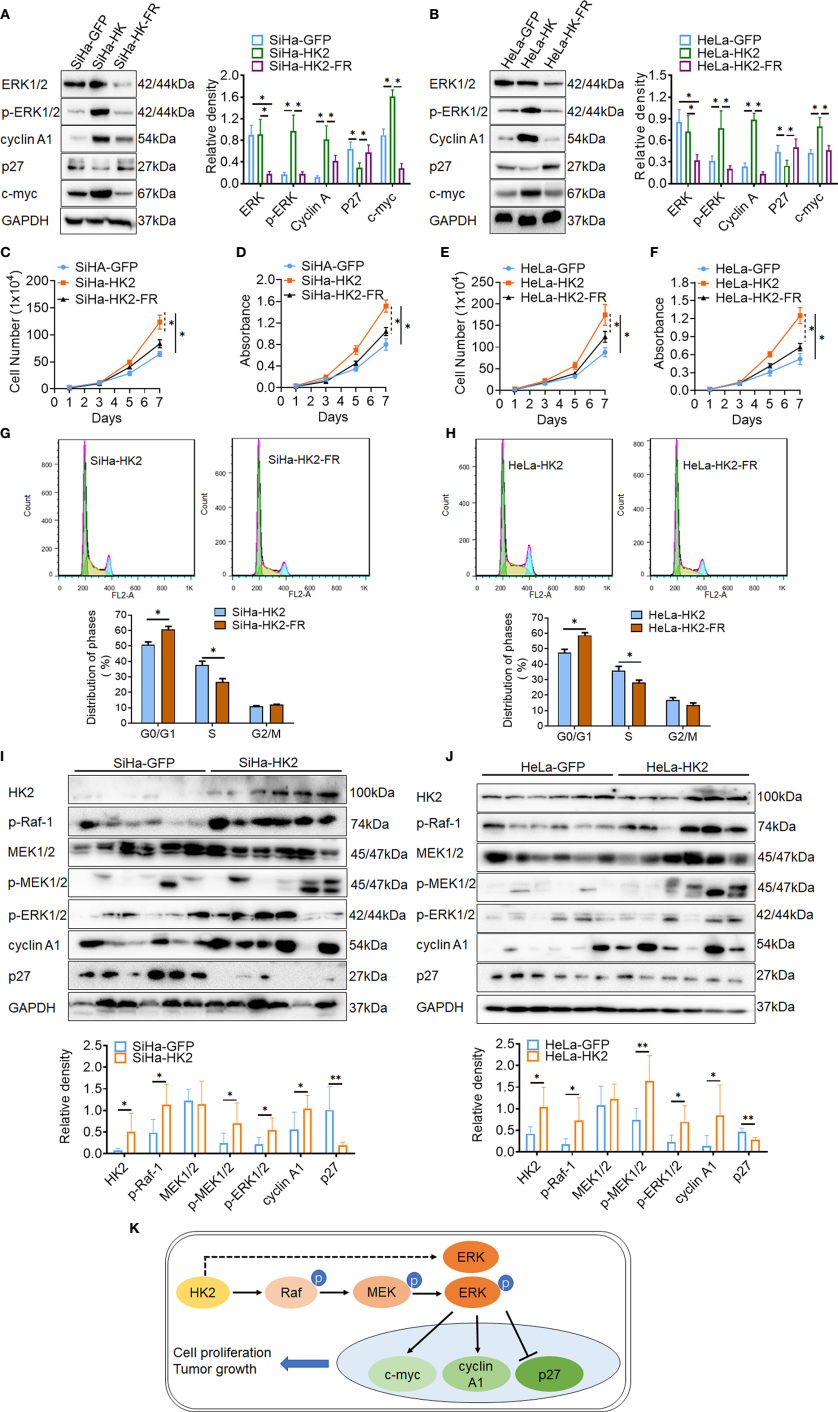
Blockage of the ERK/p-ERK expression in the HK2 over-expressed cells by using FR180204. The expression of ERK1/2, p-ERK1/2, cyclin A1, p27, and c-myc was detected by western blotting in FR180204-treated SiHa-HK2 **(A)** HeLa-HK2 **(B)** cells, and the quantitative analysis is shown. The proliferation and viability of FR180204-treated SiHa-HK2 cells were detected by growth curves **(C)** and MTT assay **(D)**. The proliferation and viability of FR180204-treated HeLa-HK2 cells were detected by growth curves **(E)** and MTT assay **(F)**. The cell cycle was analyzed in FR180204-treated HK2 overexpressed cells by using flow cytometry: **(G)** SiHa-HK2 and SiHa-HK2-FR cells and the quantitative analysis is shown; **(H)** HeLa-HK2 and HeLa-HK2-FR cells and the quantitative analysis is shown. The expression of p-Raf-1, p-MEK1/2, p-ERK1/2, cyclin A1, and p27 was detected by western blotting in xenografted tumor tissues that derived from HK2 modified cells and the quantitative analysis was shown: **(I)** SiHa-GFP and SiHa-HK2, **(J)** HeLa-GFP and HeLa-HK2 cells. **(K)** Proposed model of the HK2 promoted cell proliferation and tumor growth in cervical cancer. The data were shown as the mean ± SD of three independent experiments. *p < 0.05, **p < 0.01 *vs.* control using one-way ANOVA.

ERK activation was reported to play a fundamental role during cell cycle progression and is involved in regulating the expression of cell cycle proteins, including cyclin D1, cyclin A1, c-myc, p27, and so on (31–33). To further confirm that activated ERK1/2 was responsible for the accelerated cell cycle progress in HK2-modified cells, an inhibitor of ERK (FR180204) was used to block the expression of ERK1/2 and p-ERK1/2 in SiHa-HK2 and HeLa-HK2 cells. All of the cervical cancer cells subjected to FR180204 treatment grew much more slowly (**Figures 6C–F**, *p*<0.05) and expressed less ERK1/2, p-ERK1/2, cyclin A1, and c-myc and more p27 (**Figures 6A, B**, *p*<0.05). After treated with FR180204, a signiﬁcantly increased G0/G1 phase (50.94 ± 1.64 *vs.* 60.82 ± 1.83, *p*<0.01) and a decreased S phase (37.78 ± 2.34 *vs.* 26.67 ± 2.19, *p*<0.01) were observed in SiHa-HK2 and SiHa-HK2-FR cells. Similarly, after treated with FR180204, a signiﬁcantly increased G0/G1 phase (47.65 ± 2.04 *vs.* 58.82 ± 1.64, *p*<0.01) and a decreased S phase (35.97 ± 2.64 *vs.* 28.03 ± 1.69, *p*<0.01) were observed in HeLa-HK2 and HeLa-HK2-FR cells.

Additionally, the recombinant human EGF protein (ab9697, Abcam, USA) was used as an activator to active p-ERK expression in HK2 knockdown cells. As shown in **Supplementary Figure S3**, all of the cervical cancer cells subjected to EGF treatment expressed more p-ERK1/2, cyclin A1, and c-myc and less p27 (**Supplementary Figures S3A, B**, *p*<0.05), and grew much more quickly (**Supplementary Figures S3C–F**, *p*<0.05). After treated with EGF, a signiﬁcantly decreased G0/G1 phase (66.94 ± 2.14 *vs.* 55.52 ± 1.98, *p*<0.01) and a increased S phase (24.94 ± 1.97 *vs.* 32.03 ± 1.53, *p*<0.01) were observed in SiHa-HK2 and SiHa-shHK2-EGF cells. Similarly, after treated with EGF, a signiﬁcantly decreased G0/G1 phase (62.57 ± 2.08 *vs.* 51.57 ± 2.19, *p*<0.01) and a increased S phase (19.31 ± 1.25 *vs.* 27.59 ± 2.12, *p*<0.01) were observed in HeLa-shHK2 and HeLa-shHK2-EGF cells.

Moreover, the increased protein levels of p-Raf-1, p-MEK1/2, p-ERK1/2, and cyclin A1 and the decreased protein level of p27 were also observed in six SiHa-HK2 or HeLa-HK2 cells derived xenografted tumor tissues, compared to their control groups (SiHa-GFP and HeLa-GFP, **Figures 6G, H**, *p*<0.05). All of these results demonstrated that HK2 promoted cell proliferation and tumor formation in human cervical cancer cells by up-regulating cyclin A1, c-myc, and down-regulating p27 expression through the activation of ERK (**Figure 6I**).

## Discussion

Hexokinase (HK) catalyzes the ﬁrst committed step of glucose metabolism by converting glucose to glucose-6-phosphate (G-6P) (34). There are ﬁve hexokinase isoforms encoded by separate genes in mammalian cells. HK1 is expressed most ubiquitously in adult tissues (35). HK3 is expressed at low levels in almost all tissues (36). HK4 is expressed primarily in the liver and pancreas (37). HK5 was recently discovered but has not yet been fully characterized (38). HK2 expression is limited in most normal tissues but is frequently upregulated in various human cancers (35, 39, 40). In cervical cancer, stimulated HK2 expression was already observed back in the 1970s. Recently, immunohistochemistry analysis also showed that approximately 60% of cervical cancer specimens (n=197) stained positive for HK2. In this study, positive HK2 stains were observed in all five cervical cancer cell lines (HeLa, SiHa, C-33 A, CaSki, and HT-3), the positive rate of HK2 was increased from 25.00% (NC samples, 4/16) to 60% (HSIL samples, 9/15) and 79.49% (31/39) in SCC samples, and the relative expression level of HK2 in eight cervical carcinoma samples was also higher than that in normal cervical samples. These results were consistent with the previous studies that HK2 was stimulated in tumor samples, suggesting that HK2 expression was stimulated in cervical cancer and could be involved in the development of cervical carcinoma.

A high rate of aerobic glycolysis is a hallmark of cancers that could produce ATP molecules to supporting malignant growth. As a key mediator of aerobic glycolysis, HK2 expression is associated with the promotion of tumor metastasis and growth in many types of cancers (12). In this study, both the *in vitro* and *in vivo* experiments revealed that HK2 could promote cell proliferation and tumor formation in cervical cancer. Generally, the changes that occur during cell proliferation involve the modulation of the cell cycle. As expected, cell cycle analysis showed that HK2 accelerated cell cycle progression in both HeLa and SiHa cells. Furthermore, real-time PCR and western blot results suggested that HK2 participated in regulating the expression of two cell cycle-related proteins: cyclin A1 and p27. HK2 expression was positively correlated with cyclin A expression but negatively correlated with p27 expression in HK2-modified cells. Cyclin A1 is a cell cycle regulatory protein, playing a critical role during S-G2/M phase transition (41–43). High expression of cyclin A1 has been observed in various cancers and was correlated with proliferative activity and enhanced tumor growth (42). p27kip1 (p27) is one of cyclin-dependent inhibitors (CDKIs), playing a role as a negative regulator during cell cycle progression (44). These results indicated that this acceleratory effect of HK2 on promoting cell cycle progression in this study likely attributed to the induction of cyclin A1 and reduction of p27 expression in cervical cancer cells. Although numerous studies have shown the critical roles of cyclin A1 and p27 in regulating cell cycle progress in various cancer, however, the likely molecular regulation mechanism by which HK2 mediated cyclin A1 and p27 expression is less known.

To explore the regulation mechanism by which HK2 participated in regulating the expression of cyclin A and p27 in cervical cancer cells, a transcriptome sequencing analysis was performed to screen for potential target genes or signaling pathways potentially that mediated by HK2. Unexpectedly, *MAPK3 (ERK1)*, a key factor involving in the Raf/MEK/ERK signaling pathway (45), was identiﬁed by KEGG pathway enrichment analysis. Moreover, increased mRNA and protein levels of total ERK1/2 were observed in both SiHa-HK2 and HeLa-HK2 cells, and the opposite results were presented when endogenous HK2 expression was reduced in SiHa-shHK2 and HeLa-shHK2, suggesting that HK2 upregulates ERK1/2 expression at least at the transcriptional level. Previous studies have demonstrated that the activity of ERK was responsible for the aberrantly expression of cyclin A1 and p27 in human tumor cells (46, 47). In addition, recently study revealed that HK2 regulated cell migration, invasion, and stemness *via* FAK-ERK1/2/-MMP9-NANOG-SOX9 signaling cascades in ovarian cancer cells (48). Therefore, it was interesting to explore whether there had a potential HK2-ERK-cyclin A1/p27 signaling cascade on regulating cell proliferation and tumor formation in cervical cancer.

Nuclear translocation of ERK1/2 that participated in the biological response of cells was dependent on its phosphorylation form (p-ERK1/2), and ERK1/2 was phosphorylated by its upstream factors p-Raf/and p-MEK1/2 (49, 50). In this study, the protein levels of p-Raf-1, p-MEK1/2, ERK1/2, and ERK1/2 were increased in HK2 over-expressing cells, while total MEK1/2 expression showed no change, suggesting that ERK1/2 could be activated by simulated p-Raf-1 and p-MEK1/2 in HK2-modified SiHa and HeLa cells. Moreover, it has been known for many years that ERK activation enhances c-myc stabilization in cancer cells (51, 52). Consistently, stimulated c-myc expression was observed in HK2 over-expressing SiHa and HeLa cells, further indicating the activity of ERK in HK2 over-expressing cells.

Furthermore, to confirm the alteration of cyclin A1 and p27 was attributed to ERK1/2 activation in HK2-modified cells, an ERK inhibitor (FR180204) was used to block induced p-ERK1/2 expression in SiHa-HK2 and HeLa-HK2 cells. As shown in **Figure 6**, reduced cyclin A1 and c-myc and increased p27 expression arised when ERK1/2 and p-ERK1/2 protein was blocked in FR180204-treated HK2 over-expressing cells. In addition, using FR180204 also removed the enhanced cell growth in SiHa-HK2 and HeLa-HK2 cells that was induced by HK2. Additionally, the increased p-ERK1/2, cyclin A1 and decreased p27 protein levels were observed in xenografted tumor tissues that derived from SiHa-HK2 and HeLa-HK2 cells. All of these data indicated that ERK1/2 was activated in HK2 over-expressed cells through phosphorylated by p-Raf and p-MEK1/2 in cervical cancer cells. Otherwise, the upregulated ERK1/2 mRNA expression could also be facilitative for increased protein levels of p-ERK1/2 in SiHa-HK2 and HeLa-HK2 cells. However, further research is necessary to investigate the potential molecular regulation mechanism by which HK2 participated in upregulating ERK1/2 mRNA expression and stimulating p-Raf and p-MEK1/2 expression in cervical cancer cells. In conclusion, this study demonstrated that HK2 could activate ERK1/2 through the Raf/MEK signaling pathway, further promoting cell proliferation and tumor formation by inducing cyclin A1, c-myc and reducing p27 in cervical cancer cells.

## Data Availability Statement

Publicly available datasets were analyzed in this study. This data can be found here: https://www.ncbi.nlm.nih.gov/bioproject/670405.

## Ethics Statement

The studies involving human participants were reviewed and approved by the Ethics Committee of the First Affiliated Hospital of the Medical School of Xi ‘an Jiaotong University. The patients/participants provided their written informed consent to participate in this study.

## Author Contributions

NC and LL designed this paper. QF, H-MM, DL, and LL performed the experiments. NC and P-SZ wrote this paper and analyzed the data. All authors contributed to the article and approved the submitted version.

## Funding

This work was supported by a grant of P-SZ from the National Natural Science Foundation of China (No. 81672910) and NC from the National Natural Science Foundation of China (No. 81903042).

## Conflict of Interest

The authors declare that the research was conducted in the absence of any commercial or financial relationships that could be construed as a potential conflict of interest.
