# Supplementary material for: Hexokinase 2 Promotes Cell Growth and Tumor Formation Through the Raf/MEK/ERK Signaling Pathway in Cervical Cancer
Source: Front Oncol. 2020 Nov 26;10:581208. doi: 10.3389/fonc.2020.581208 (PMC7725710; doi:10.3389/fonc.2020.581208)
Supplement: Supplementary file 1 [file DataSheet_1.doc]

**Supporting Information**

**
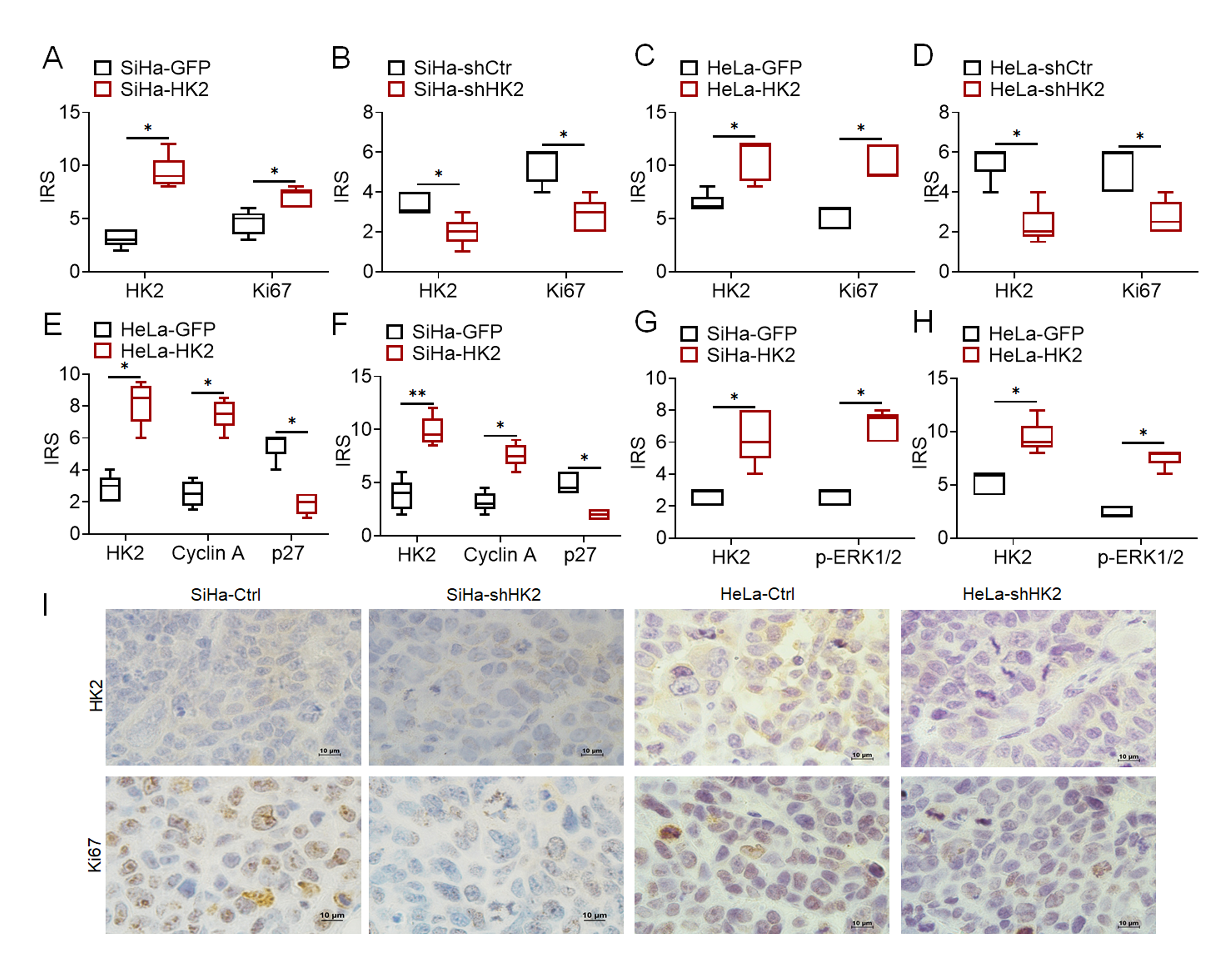
**

**Figure S1: The quantitative analysis for immunohistochemical and immunocychemistry stains**

The quantitative analysis for immunohistochemical stains of HK2 and Ki67 in xenograft tumor tissues: (A) SiHa-GFP, SiHa-HK2 cells, (B) SiHa-shControl and SiHa-shHK2 cells; (C) HeLa-GFP, HeLa-HK2 cells, (D) HeLa-shControl and HeLa-shHK2 cells. (E) The quantitative analysis for immunocychemistry stains of HK2, cyclin A1 and p27 in SiHa-GFP and SiHa-HK2 cells. (F) The quantitative analysis for immunocychemistry stains of HK2, cyclin A1 and p27 in HeLa-GFP and HeLa-HK2 cells. (G) The quantitative analysis for immunocychemistry stains of HK2 and p-ERK1/2 in SiHa-GFP and SiHa-HK2 cells. (H) The quantitative analysis for immunocychemistry stains of HK2 and p-ERK1/2 in HeLa-GFP and HeLa-HK2 cells.

(I) Immunohistochemical staining of HK2 and Ki67 in SiHa-shHK2 and HeLa-shHK2 cells drived xenograft tumor tissues. The data were shown as the mean±SD of three independent experiments. ** p<0.05, ** p<0.01 vs.* control using One-Way ANOVA.

**
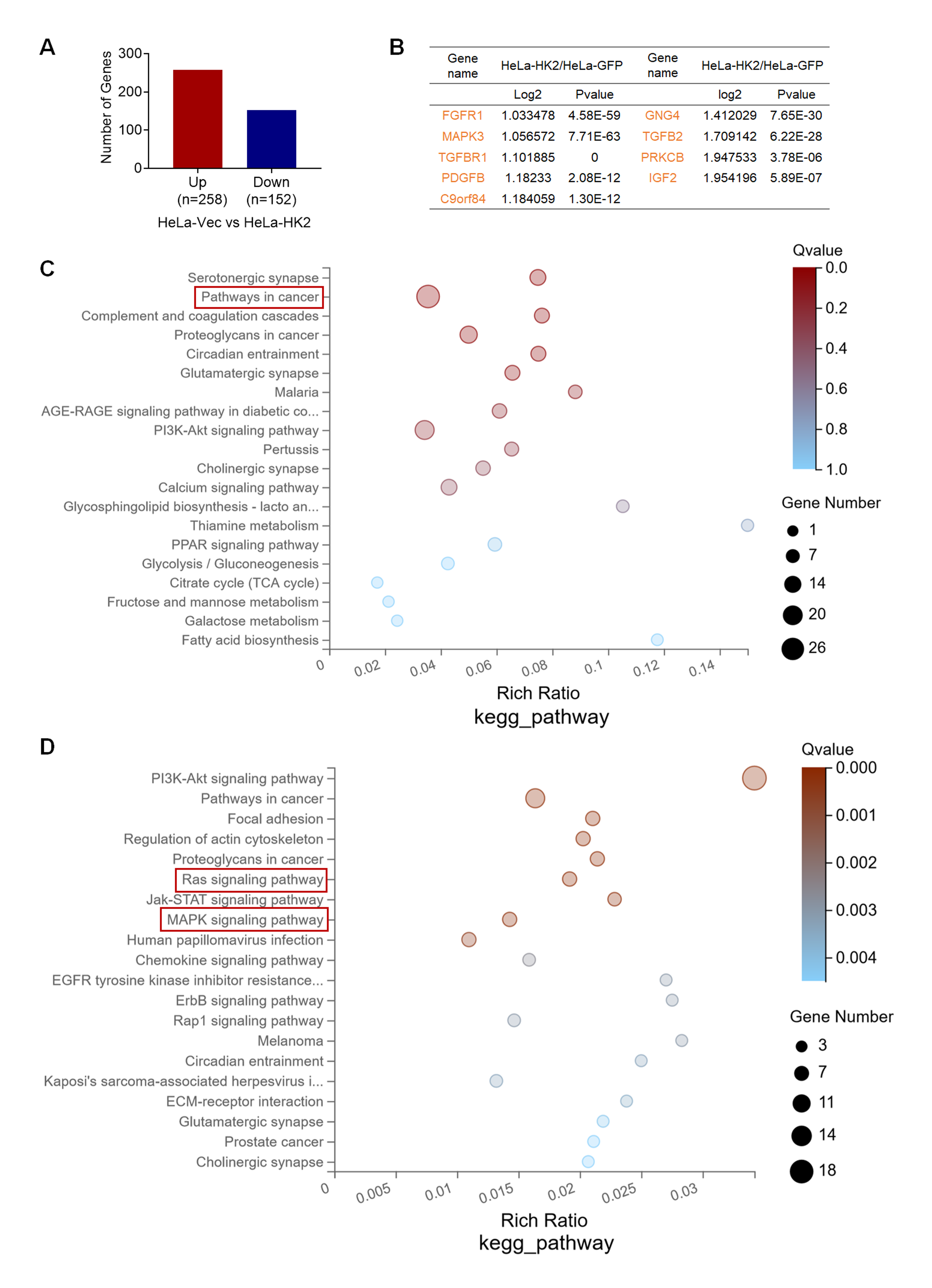
**

**Figure S2: The transcriptome sequencing analysis in HeLa-HK2 and HeLa-GFP monoclonal cell lines.**

(A) Total of 258 up-regulated and 152 downregulated genes was identiﬁed between HeLa-HK2 and HeLa-GFP groups by using the transcriptome sequencing analysis. (B) The gene list of 9 genes that belonged to Ras/MAPK signaling pathway was identiﬁed by Gene Ontology (GO) enrichment analysis. (C) Gene Ontology (GO) enrichment analysis identiﬁed the expression of 26 genes that involved in the pathway in cancer was signiﬁcantly changed between HeLa-HK2 and HeLa-GFP groups. (D) KEGG Pathway enrichment analysis identiﬁed that there are 9 genes belonged to Ras/MAPK signaling pathway and 12 genes belonged to PI3K-Akt signaling pathway.

**
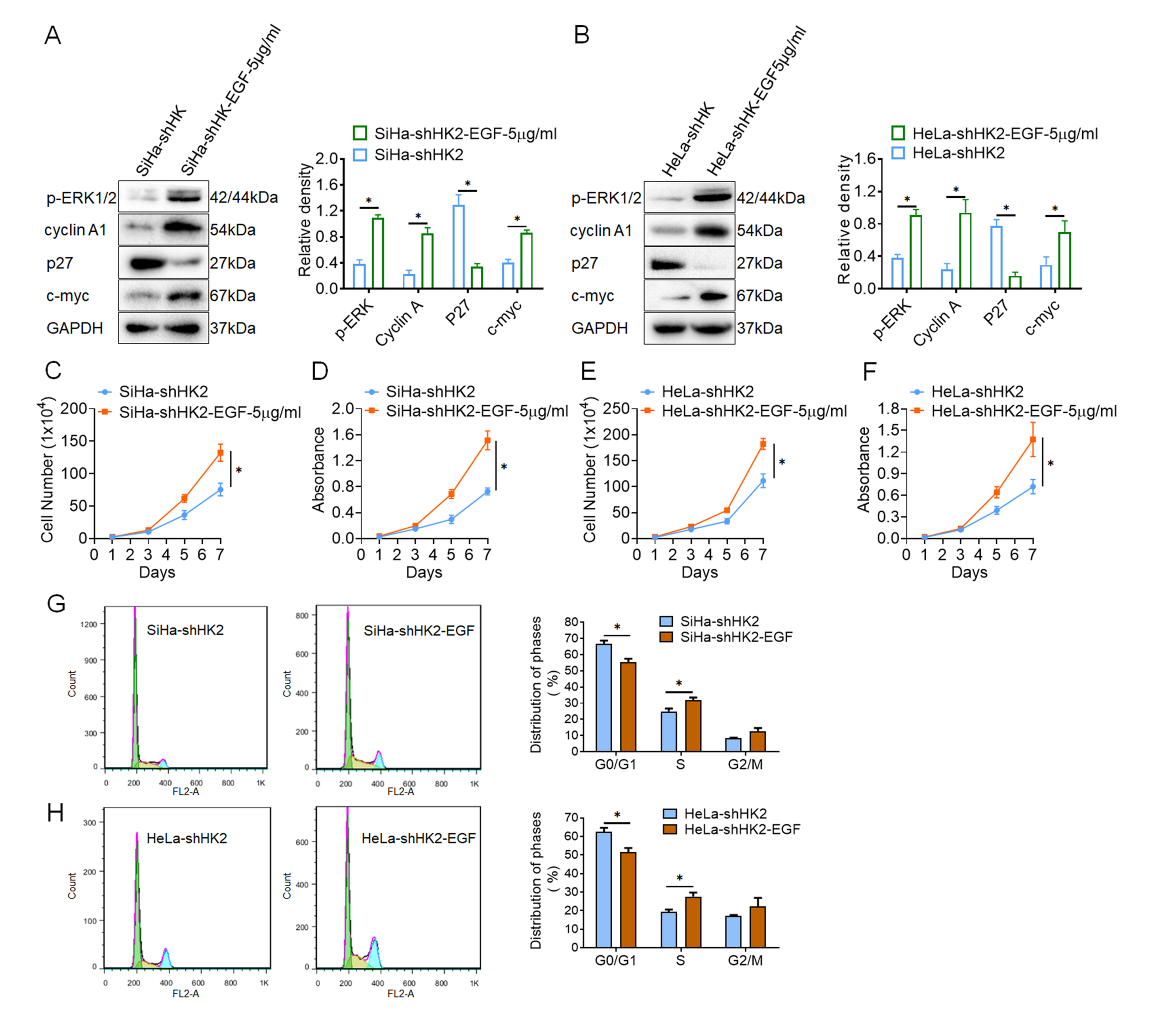
**

**Figure S3: Rescue of the p-ERK expression in the HK2 knockdown cells by using the recombinant human EGF protein**

The expression of p-ERK1/2, cyclin A1, p27 and c-myc was detected by western blotting in EGF-treated (5µg/ml, treated for 24 h) SiHa-shHK2 (A) and HeLa-shHK2 (B) cells, and the quantitative analysis is shown. The proliferation and viability of EGF-treated SiHa-shHK2 cells were detected by growth curves (C) and MTT assay (D). The proliferation and viability of EGF-treated HeLa-shHK2 cells were detected by growth curves (E) and MTT assay (F). The cell cycle was analyzed in EGF-treated HK2 knockdown cells by using flow cytometry: (G) SiHa-shHK2 and SiHa-shHK2-EGF cells and the quantitative analysis is shown; (H) HeLa-shHK2 and HeLa-shHK2-EGF cells and the quantitative analysis is shown.
