# Supplementary material for: Hexokinase 2 Promotes Cell Growth and Tumor Formation Through the Raf/MEK/ERK Signaling Pathway in Cervical Cancer
Source: Front Oncol. 2020 Nov 26;10:581208. doi: 10.3389/fonc.2020.581208 (PMC7725710; doi:10.3389/fonc.2020.581208)
Supplement: Supplementary file 2 [file Table_1.doc]

**Supporting Information**

Abbreviation: NC: normal human cervix, HSIL: high-grade squamous intraepithelial lesion, SCC: squamous cervical cancer

| **Table S1.** HK2 Expression Levels in Different Tissue Specimen | | | | |
| --- | --- | --- | --- | --- |
| **Specimens** | **Total** | **Slug Staining** | | **P** |
|  |  | **Negative, No. (%)** | **Positive, No. (%)** |  |
| NC | 16 | 12(80.00) | 4(20.00) |  |
| HSIL | 15 | 6(40.00) | 9(60.00) | 0.07^a^ |
| SCC | 39 | 17(20.51) | 31(79.49) | <0.01^b^ |

Pearson 2-tailed chi-square test was used to determine the statistical significance of the level of expression of HK2 in different tissue specimens.

a: NC vs HSIL

b: NC vs SCC

**Table S2.** **The list of primer sequences that used for real time PCR** in this study.

| **Primer name** | **F/R** | **Sequence** |
| --- | --- | --- |
| **Cyclin A1** | F | GAGGTCCCGATGCTTGTCAG |
|  | R | GTTAGCAGCCCTAGCACTGTC |
| **Cyclin D1** | F | GCTGCGAAGTGGAAACCATC |
|  | R | CCTCCTTCTGCACACATTTGAA |
| **Cyclin E1** | F | AAGGAGCGGGACACCATGA |
|  | R | ACGGTCACGTTTGCCTTCC |
| **P21** | F | TGTCCGTCAGAACCCATGC |
|  | R | AAAGTCGAAGTTCCATCGCTC |
| **P27** | F | AACGTGCGAGTGTCTAACGG |
|  | R | CCCTCTAGGGGTTTGTGATTCT |
| **C-MYC** | F | GGCTCCTGGCAAAAGGTCA |
|  | R | CTGCGTAGTTGTGCTGATGT |
| **HK2** | F | GAGCCACCACTCACCCTACT |
|  | R | CCAGGCATTCGGCAATGTG |
| **ERK1** | F | CTACACGCAGTTGCAGTACAT |
|  | R | CAGCAGGATCTGGATCTCCC |
| **ERK2** | F | TACACCAACCTCTCGTACATCG |
|  | R | CATGTCTGAAGCGCAGTAAGATT |
| **GAPDH** | F | GCACCGTCAAGGCTGAGAAC |
|  | R | TGGTGAAGACGCCAGTGGA |
